# Concurrent ipsilateral Tillaux fracture and medial malleolar fracture in adolescents: management and outcome

**DOI:** 10.1186/s13018-020-01961-7

**Published:** 2020-09-17

**Authors:** Quanwen Yuan, Zhixiong Guo, Xiaodong Wang, Jin Dai, Fuyong Zhang, Jianfeng Fang, Chunhua Yin, Wentao Yu, Yunfang Zhen

**Affiliations:** Children’s Hospital of Soochow University, No. 92 Zhongnan street, Suzhou, Industrial Park, Jiangsu China

**Keywords:** Adolescents, Ankle, Mallelous, Tibia, Tillaux fracture

## Abstract

**Background:**

The concurrent ipsilateral Tillaux fracture with medial malleolar fracture in adolescents commonly suffer from high-energy injury, making treatment more difficult. The aim of this study was to discuss the mechanism on injury, diagnosis, and treatment of this complex fracture pattern.

**Methods:**

The charts and radiographs of six patients were reviewed. The function was assessed by the American Orthopedic Foot and Ankle Society ankle-hindfoot scores.

**Results:**

The mean age at operation was 12.8 years. The mean interval from injury to operation was 7.7 days. Five Tillaux fractures and all medial malleolar fractures were shown on AP plain radiographs. One Tillaux fracture and two cases with avulsion of posterolateral tibial aspect were confirmed in axial computerized tomography. There was talar subluxation laterally with medial space widening in three and syndesmotic disruption in one. There were five patients sustaining ipsilateral distal fibular fractures. All fractures, except nonunion in two medial malleolar fractures and in one Tillaux fracture, healed within 6–8 weeks. There was one case of osteoarthritis of ankle joint. The average AOFAS score was 88.7.

**Conclusions:**

Computerized tomography is helpful in identifying the fracture pattern. Anatomic reduction and internal fixation of Tillaux and medial malleolar fracture was recommended to restore the articular surface congruity and ankle stability.

## Background

Physeal injuries of the distal tibia are second in frequency to those of the distal radius and carry a high risk of complications. Tillaux fracture accounts for approximately 2.9–6.7% of the distal tibial epiphyseal fractures [1, 2]. It usually occurs in adolescents when the center and medial side of the distal tibial physis have been closed and the anterolateral quadrant fusion does not occur. The mechanism of injury involved external rotation of the foot contributing to the avulsion of the anterior inferior tibiofibular ligament (AITFL). The pediatric medial malleolar fractures (MMF) usually involved the growth plate, often Salter-Harris III or IV fractures, and carried the highest risk of complication for the premature physeal closure (PPC) [3–5], whereas fractures of other parts of the medial malleolus have received sporadic attention in the literature for lower rate of growth disturbance and non-weight-bearing area. However, the medial malleolus plays an important role in ankle stability as a bony restraint [6].

Isolated fractures, aforementioned, are usually suffering from indirect force with particular foot position. Direct or high-energy trauma to the ankle may result in special injury configurations. Recently, Gourineni and Gupta [7] reported that six out of eight Tillaux fractures had associated with talar subluxation (TS), lateral malleolar fractures. To our knowledge, there has been no report regarding to the combination of Tillaux fracture and MMF in adolescents. It was the purpose of this study to report the experience of a pediatric trauma center in an attempt to discuss its mechanism of injury, diagnosis, and treatment options.

## Methods

This study was approved by the ethics committee of the Children’s Hospital of Soochow University. Six cases of Tillaux fracture associated with MMF were treated from January 2015 to December 2018. The charts and radiographs of six patients were reviewed retrospectively. Clinical data, including age, sex, mechanism of injury, and methods of treatment, were collected (Table 1). There were three boys and three girls, with a mean age at operation of 12.8 years (range, 12 to 14 years). The left side was involved in three and the right in three. One patient was hurt while falling down, three during sports of sliding or soccer, and two in a motor vehicle accident. The mean follow-up was 11.7 months (range, 6 to 27 months).

**Table 1:** Patient demographic data

No.	Gender	Age (years + months)	Interval (days)	Lateral	Cause	Associated injuries	TMM	DT (mm)	SD	Treatment	Complications	Follow-up(months)	AOFAS
1	F	14 + 11	3	L	Traffic	_	S-H III	5.6		ORIF	—	6	96
2	F	12 + 9	6	R	Sliding	Distal fibular Fx; TS	H-B	8	No	ORIF P + S	—	13	90
3	M	14 + 11	5	L	Traffic	Distal fibular Fx	H-C	2	No	ORIF	—	8	96
4	F	12 + 1	5	R	Sliding	Distal fibular Fx, avulsion of PLTA, TS	H-C	9.4	No	ORIF P + S	Nonunion of medial malleoli	10	90
5	M	13 + 1	20	R	Falling	Distal fibular and S-H I epiphyseal Fx, TS	H-C	8.2	Yes	Traction P + S	Nonunion of Tillaux and medial malleoli, instability, OA	27	64
6	M	12 + 10	7	L	Soccer	Distal fibula Fx, avulsion of PLTA	H-C	8.8	No	ORIF	—	6	96

*Fx* fracture, *TS* talar subluxation, *PLTA* posterolateral tibial aspect, *ORIF* open reduction and internal fixation for Tillaux and/or medial malleolar fractures, *P + S* plate + screws for distal fibular fracture, *TMM* type of medial malleolar fracture, *DT* initial displacement of Tillaux fragment, *SD* syndesmotic disruption, *OA* osteoarthritis

Anteroposterior (AP), lateral plain radiographs and computerized tomography (CT) were performed in all patients to identify the fractures. Axial CT views were reviewed for displacement of Tillaux fractures in the maximal gap section in millimeters and to identify the inferior tibiofibular syndesmosis disruption (SD) and the avulsion of posterior inferior tibiofibular ligament at tibial attachment (APITEL).

MMF were classified according to Salter-Harris epiphyseal injury (S-H) when the physis was involved [8]. Otherwise, Herscovici classification (H), based on the level of MMF, was used: type A, fracture being avulsions of the tip; type B, between the tip and the level of the plafond; type C, at the level of the distal plafond of the tibia; type D, extended vertically above the plafond [6]. TS was considered if medial joint space of the ankle was greater than the superior ankle space in AP plain radiographs or coronal CT scans [7].

For Tillaux fracture, open reduction was performed through an anterolateral ankle approach, and anatomical reduction was obtained and maintained by Kirschner wires (K-wires). Closed reduction and K-wire fixation were undergone for MMF. If necessary, open reduction and internal fixation (ORIF) of the fibular fracture was done with plate and screws. Postoperatively, a short-leg cast was applied, and the patients were instructed not to bear weight for 6–8 weeks.

Functional assessment was by American Orthopedic Foot and Ankle Society (AOFAS) ankle-hindfoot scores [9].

## Results

Five Tillaux fractures and all MMF were shown on AP radiographs. There were five patients sustaining ipsilateral distal fibular fractures. One Tillaux fracture and two APITELs were confirmed in axial CT. All Tillaux fragments were rotated laterally, and the mean initial displacement was 7 mm (range, 2.0 to 9.4 mm) in axial CT. Out of six MMFs, one was S-H III fractures, one H-type B, and four H-type C. Avulsion in both PLTA cases had minimal displacement. AP radiographs showed TS laterally with medial space widening in three patients, and axial CT showed SD in one case.

The mean interval from injury to operation was 7.7 days (range, 3 to 20 days). For Tillaux fracture, five cases were treated with ORIF with K-wires, and one without internal fixation owing to 20 days interval after injury for significant soft tissue compromise. For MMF, open reduction and K-wire fixation were underwent in S-H III fracture, five closed reduction, and K-wire fixation for H-types B and C. One failed to be fixed due to technique error. Of five fibular fractures, open reduction and fixation with plate and screws were needed to stabilize the ankle joint in three cases with TS laterally. Syndesmotic fixation was not used for SD, and neither of the avulsion of PLTA was fixed for small fragment and minimal displacement.

All fractures, except two cases of nonunion in MMF and one in Tillaux fracture, healed within 6–8 weeks. Radiographs of the child without reduction of Tillaux and MMF (case 5) demonstrated a valgus of ankle joint, hindfoot instability, and radiographic sign of osteoarthritis. Neither PPC nor leg length discrepancy developed in any of the patients.

**Fig. 1:**
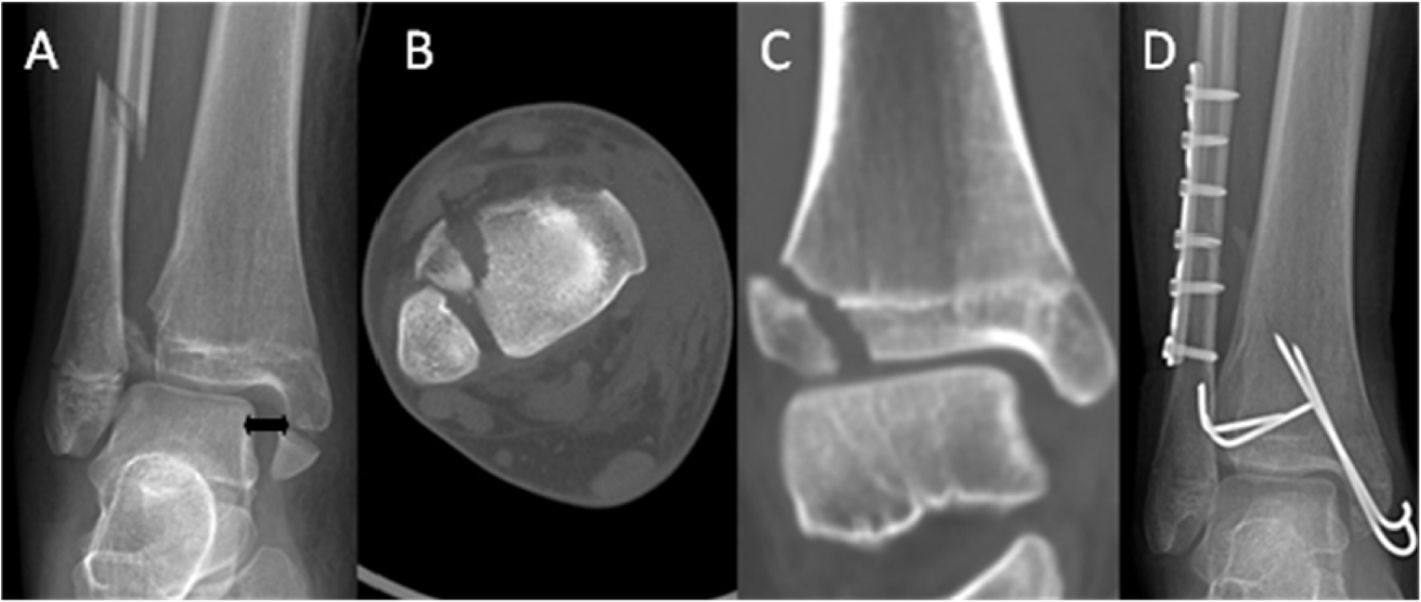
(Case 2): **a** AP radiograph showed Tillaux fracture, distal fibular fracture, and MMF associated with widening of the tibiofibular clear space and medial space of the ankle (black double arrow). **b** Axial CT section showed 8 mm displacement of Tillaux fragment and a normal incisura fibularis. **c** Coronal CT scan showed widening of the medial clear space of the ankle, which indicates TS. **d** AP plain radiograph obtained 12 months after treatment showed complete reduction of medial joint space immediately post operatively

**Fig. 2:**
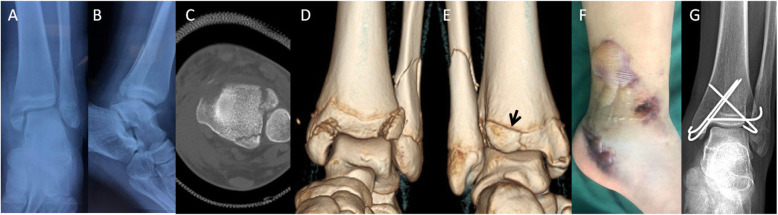
(Case 6): **a** AP radiograph showed distal fibular fracture and MMF associated. **b** Lateral view showed a radiolucent zone consistent with a fracture at the posterior aspect of the tibia, but it was not clear because of superimposition of the fibula. **c**, **d**, and **e** Axial CT section and 3D showed Tillaux fragment and APITEL (black arrow). **f** General observation showed significant edema of the soft tissue. **g** AP plain radiograph obtained 7 months after treatment showed complete reduction of medial joint space immediately post operatively

The AOFAS score was 88.7 (64 to 97) at the final visit (Table 1). Typical cases are shown in Figs. 1 and 2.

## Discussion

Tillaux fracture and medial malleolar fracture are usual fractures in children. However, Tillaux fracture associated with medial malleolar fracture are rare in children. We described a fracture type of Tillaux fracture concomitant with medial malleolar fracture. This may be due to sufficient external rotation force. Tillaux fragment, talus, and lateral malleolus being one unit will move laterally together, and the fracture of distal fibula and APITEL will occur. Extreme external rotation of the talus may disrupt the medial restraint and create MMF in adolescents [7, 10]. Due to the high-energy force, the displacement of the fracture, such as Tillaux fracture and medial malleolar fracture, was more than isolated fracture. In this series, three patients suffered from TS with widening of medial joint space and one with SD.

In our experience, anatomical reduction for Tillaux fracture and medial malleolar fracture was restored, and the fracture was fixed by K-wires. The result was comparable with the fixation of screws [7, 11]. There were two nonunion in medial malleolar fracture cases because of no internal fixation. Many factors were related with the bone healing [12, 13]. Our results suggest that internal fixation is more effective for MMF in adolescents. For distal fibular fracture, fixation was performed only with TS. For APITEL in adolescents, we found no displacement and no fixation were undergone for both cases.

This study is limited by small group and relative shorter follow up period. More cases should be necessary to confirm the mechanism of injury and optimal treatment.

## Data Availability

All data generated or analyzed during this study are included in this manuscript.

## Abbreviations

AITFL: Anterior inferior tibiofibular ligament
MMF: Medial malleolar fracture
PPC: Premature physeal closure
S-H: Salter-Harris epiphyseal injury type
H: Herscovici classification
AP: Anteroposterior
CT: Computed tomography
APITEL: Avulsion of posterior inferior tibiofibular ligament
K-wire: Kirschner wire
TS: Talar subluxation
SD: Syndesmotic disruption
ORIF: Open reduction and internal fixation
